# Clinico-pathological profile of head and neck malignancies at University College Hospital, Ibadan, Nigeria

**DOI:** 10.1186/1746-160X-7-9

**Published:** 2011-05-13

**Authors:** Akinyele O Adisa, Bukola F Adeyemi, Abideen O Oluwasola, Bamidele Kolude, Effiong EU Akang, Jonathan O Lawoyin

**Affiliations:** 1 Departments of Oral Pathology University College Hospital, University of Ibadan, Ibadan, Oyo state, Nigeria; 2 Department of Pathology, University College Hospital, University of Ibadan, Ibadan, Oyo state, Nigeria

**Keywords:** head and neck malignancies, clinico-pathologic profile, south-western Nigeria

## Abstract

**Introduction:**

This retrospective study analysed head and neck malignancies seen over a 19-year period at the University College Hospital, Ibadan.

**Methodology:**

One thousand, one hundred and ninety two patients with head and neck malignancies were analysed according to age, gender, topography and histology.

**Results:**

There was an annual hospital frequency of 62 cases per year. The overall mean age for these malignancies was 43.9 (SD ± 19.3) years. The lesions from the respiratory tract were the most frequent (43.2%) of all cases. The palate was the most frequent intra-oral site (13.8%). Epithelial malignancies constituted 73.4% of all cases with a male: female ratio of 2:1, a mean age of 48.1 (SD ± 17.5) years and were mostly located in the larynx (19.7%). Lymphomas constituted 17.5% of all head and neck cancers with a male: female ratio of 1.6:1, a mean age of 35.1 (SD ± 20.6) years and nodal involvement (39.7%) was most common. Sarcomas constituted 8.9% of all malignancies with a male: female ratio of 1.5:1, mean age of 27.1 (SD ± 16.7) years and the maxillofacial bones (42.5%) were most commonly involved. Neuroendocrine malignancies accounted for 0.2% of head and neck malignancies with a male: female ratio of 1:1, a mean age of 28.5 (SD ± 6.4) years and both cases involved the nose.

**Conclusion:**

This study has further confirmed that carcinomas remain the most frequent cancers of the head and neck region in south-western Nigeria.

## Introduction

Head and neck cancers are malignant neoplasms occurring in the nasal cavities, paranasal sinuses, nasopharynx, hypopharnyx, oropharynx, ear, scalp, oral cavity and salivary glands [1]. These malignancies are associated with various aetiological factors such as tobacco and alcohol use [2], infection by oncogenic viruses, genetic factors and nutritional deficiency [3].

Head and neck cancer is the tenth most common cancer in the world [4] and is an important cause of morbidity and mortality [5]. Patients with head and neck cancer have specific requirements that are beyond the needs of most other patients diagnosed with other types of cancer [6]. Several assorted histological types of tumours are found in the head and neck region. Between 70% to 90% of head and neck cancers are epithelial in origin, and squamous cell carcinoma constitutes 66.7% of carcinomas and 47.8% of all head and neck cancers [7,8]. About 30% of all lymphomas occur in this region and they comprise the second most common primary malignancy in the head and neck region [9]. About 15% to 20% of all sarcomas are diagnosed in the head and neck region [10]. Osteogenic sarcoma, rhabdomyosarcoma, malignant fibrous histiocytoma and angiosarcoma are the most common histological types [11]. Salivary gland malignancies constitute about 1% of all head and neck cancer [7].

The prospects of head and neck cancer depends on histological type, degree of histological differentiation of the tumour cells, clinical staging, primary site of tumour, age of patient, co-morbid conditions and neuro-vascular invasion [12]. The purpose of this study therefore is to estimate the importance of a clinico-pathological profile of head and neck malignancies in western Nigeria.

## Methodology

This is a retrospective study that provides analysis of head and neck malignancies (with respect to age, gender, topography and histological diagnosis) at the University College Hospital (U.C.H.) Ibadan from January 1990 to December 2008. Local ethical clearance was obtained from the Joint University of Ibadan/University College Hospital Ethical Review Committee (registration number: NHREC/05/01/2008a).

Biopsy report registers were obtained from the departments of Oral Pathology and Pathology U.C.H Ibadan and records of all malignant lesions involving the oral and nasal cavities, the paranasal sinuses, oropharynx, nasopharynx, hypopharynx, larynx, trachea, ear and salivary glands [1] were included. Malignancies involving the thyroid, eye and brain were excluded [1].

The age grouping system used is that recommended for morbidity in health by the Department of International Economic and Social Affairs of the United Nations [13]. 1-14 years (children), 15-24 years (young adults or adolescents), 25-44 years (older adults), 45-64 years (middle aged), ≥65 years (elderly).

The data was entered into the version 16 of the Statistical Package for Social Sciences (SPSS16). Qualitative data were expressed as percentages and compared using chi-square statistics. Quantitative data were summarised using mean, standard deviation and confidence interval. The data were further compared using student t-test and/or one-way analysis of variance test as appropriate. The level of significance was set at p < 0.05

## Results

**Figure 1:**
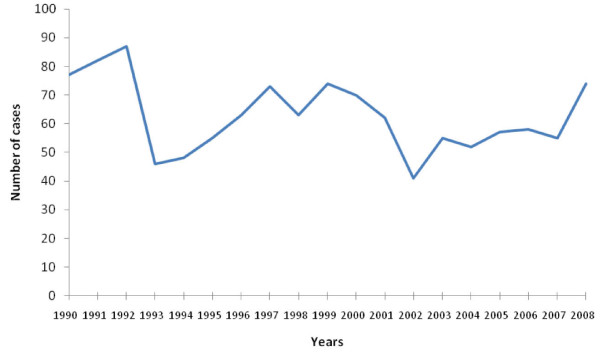
**Annual frequency of head and neck malignancies**.

The hospital based prevalence was about 62 cases of malignant head and neck neoplasms per year. The time trend of relative frequencies during the period showed no regular pattern. However, none of the years recorded less than 40 patients (Figure 1).

### Gender Distribution

A total of 781 (65.5%) males and 410 (34.4%) females presented during the period under study [the gender of 1 person (0.1%) was not indicated in the record]. The overall approximate male to female ratio for malignant head and neck neoplasms was 1.9:1.

### Age Distribution

**Figure 2:**
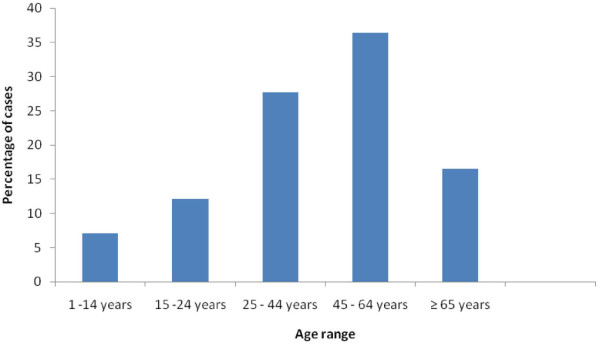
**Age distribution of head and neck malignancies**.

The patients' ages ranged from 1 year to 98 years with a mean of 43.9 years (SD ± 19.3). There was no statistically significant difference between the mean ages of males and females (t = 1.145, df = 1187, p = 0.253). Head and neck malignancies occurred least frequently in the first decade of life and displayed a gradual increase until it peaked in the 45-64 age range (36.4% of cases) after which the incidence declined (Figure 2).

### Topography (general)

The topographical distribution of the head and neck malignancies generally showed that lesions arising from the respiratory tract were the most frequent, accounting for 43.2% of the cases (Table 1). These are lesions of the nose, nasopharynx, oropharynx, hypopharynx and larynx. Other less common sites included the maxillofacial bones (20.5%), oral cavity (12.5%) and cervical lymph nodes (11.2%).

**Table 1:** Anatomical distribution of head and neck malignancies

ANATOMICAL LOCATION	FREQUENCY	PERCENT
**Respiratory tract**	**516**	**43.2**
**Maxillofacial bones**	**244**	**20.5**
**Intraoral**	**149**	**12.5**
**Cervical lymph nodes**	**133**	**11.2**
**Salivary Glands**	**64**	**5.4**
**Face and scalp (soft tissue)**	**63**	**5.3**
**Ear**	**16**	**1.3**
**Oesophageal**	**7**	**0.6**
**TOTAL**	**1192**	**100**

*maxillofacial bones = maxilla, mandible, skull, maxillary sinus

### Topography of malignant maxillofacial tumours

The maxilla (24.3%) represented the most frequent site of occurrence in the oro-facial complex, followed by the mandible (19.7%) and the salivary glands (13.9%), as shown in Table 2. Intraorally, however the palate was found to be the most frequently affected.

**Table 2:** Anatomical distribution of malignant maxillofacial tumours

SITE	FREQUENCY	LOCAL PERCENTAGE	OVERALL PERCENTAGE
**Maxilla**	105	24.2	8.8
**Mandible**	85	19.6	7.1
**Salivary glands**	60	13.9	5.0
**Palate**	55	12.7	4.6
**Tonsil**	26	6.0	2.2
**Cheek**	25	5.8	2.1
**Tongue**	25	5.8	2.1
**Floor of mouth**	11	2.5	0.9
**Lip**	9	2.1	0.8
**Face**	32	7.4	2.7
**TOTAL**	433	100	

### Broad Histological Types

**Figure 3:**
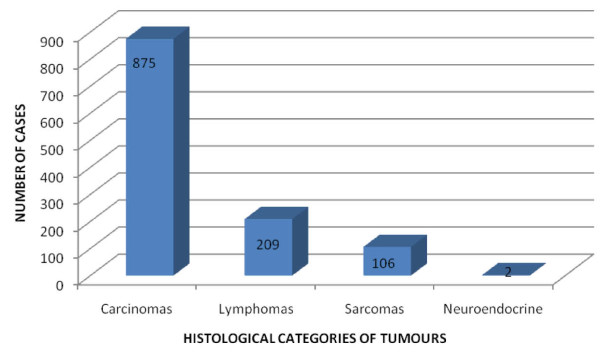
**Broad histological types of head and neck malignancies**.

The epithelial malignancies constituted 73.4% (875 patients) of all the cases (figure 3). Lymphomas and sarcomas constituted 17.5% and 8.9% of cases, respectively. There were two neuroendocrine tumours, accounting for 0.2% of the cases.

### Trends of the histological diagnosis

**Figure 4:**
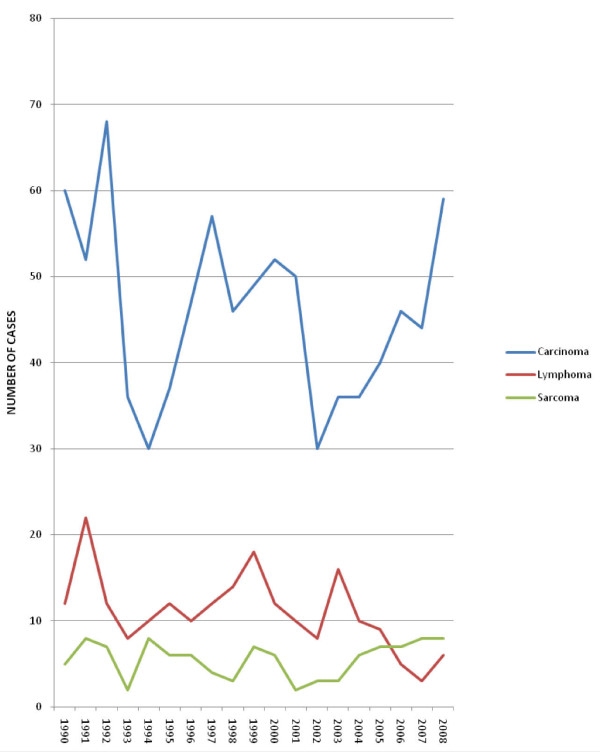
**Annual frequency of broad histological types of head and neck malignancies**.

Diagnosis of carcinomas exceeded (at least by a factor of 3) any other type of malignant lesion, every year consistently for 19 years. A sustained increase in the prevalence of carcinomas was also noted from 2002 to 2008, while lymphoma cases reduced comparatively from 2004 to 2007 (figure 4).

### Gender and age distribution within the broad histological types

The male to female gender ratio for the broad histological types was 2:1 for carcinomas, 1.5: 1 for sarcomas, 1.6:1 for lymphomas and 1:1 for neuroendocrine tumours (table 3).

**Table 3:** Gender distribution of broad histological types of head and neck cancer

	CARCINOMAS	SARCOMAS	LYMPHOMAS	NEUROENDOCRINE CARCINOMA	TOTAL
**MALE**	586 (67.0%)	65 (61.3%)	129 (61.7%)	1 (50%)	781
**FEMALE**	288 (32.9%)	41 (38.7%)	80 (38.3%)	1 (50%)	410
**MISSING**	1 (0.1%)				1
**TOTAL**	875 (100%)	106 (100%)	209 (100%)	2 (100%)	1192

Most cases of carcinomas were diagnosed in the 45-64 age group, with 361 (41.3%) patients (Table 4). The sarcomas were most prevalent in the 25-44 years age range with 35 (33.0%) patients, closely followed by age group 15-24 years with 34 (32.1%) patients. A total of 85 head and neck malignancies were found in the age group 1-14 years with the most common lesion being lymphomas, which had 46 (54.1%) patients. Sarcomas were the second most common, consisting of 20 (23.5%) patients. Carcinomas accounted for 19 cases (22.3%), while there were no neuroendocrine tumours in the 1-14 year age group (Table 4).

**Table 4:** Age distribution of broad histological types of head and neck cancer

**HISTOLOGICAL TYPES**	**AGE GROUP (years)**					
	
	**1-14**	**15-24**	**25-44**	**45-64**	**≥65**	**TOTAL**
**Carcinomas**	19(2.2%)	77(8.8%)	238(27.2%)	361(41.4%)	178(20.4%)	873*(100%)
**Lymphomas**	46(22%)	32(15.3%)	56(26.8%)	59(28.2%)	60(28.7%)	209(100%)
**Sarcomas**	20(18.9%)	34(32.1%)	35(33%)	14(13.2%)	3(2.8%)	106(100%)
**Neuroendocrine tumours**	0	1(50%)	1(50%)	0	0	2(100%)
**TOTAL**	85(7.1%)	144(12.1%)	330(27.7%)	434(36.5%)	197(16.6%)	1190(100%)

*Two ages missing

Grouping the malignant lesions into their broad lineages the mean ages of those with carcinomas, sarcomas, lymphomas and neuroendocrine tumours were 48.1 (SD ± 17.5), 27.1 (SD ± 16.7), 35.1 (SD ± 20.6) and 28.5 (SD ± 6.4) respectively.

### Topographic distribution of the broad histological types of head and neck cancer

The predominant anatomical sites for carcinomas were the larynx, nasopharynx, maxillofacial bones and oral cavity, in descending order of frequency (Table 5). Lymphomas were most frequent in the lymph nodes (39.7%) followed by the maxillofacial bones. By contrast, sarcomas occurred most frequently in the maxillofacial bones (42.5%), face/scalp (17.9%) and the nose (12.3%). Both cases of the neuroendocrine carcinomas occurred in the nose (Table 5).

**Table 5:** Anatomical distribution of broad histological types of head and neck cancer

Histological type	N	NP	OP	HP	LRX	EO	EAR	IO	MB	F/S	LN	SG	Total
**Carcinomas**	93	164	9	6	172	7	14	125	144	40	41	60	875
**Lymphomas**	20	21	4	0	1	0	1	17	55	4	83	3	209
**Sarcomas**	13	6	1	1	3	0	1	7	45	19	9	1	106
**Neuroendocrine**	2	0	0	0	0	0	0	0	0	0	0	0	2
**TOTAL**	128	191	14	7	176	7	16	149	244	63	133	64	1192

KEY N-Nose, NP-nasopharynx, OP-oropharynx, HP-hypopharynx, LRX-larynx, EO-oesophagus, IO-intraoral, MB-maxillofacial bones, FS-face and scalp, LN-cervical lymph nodes, SG-salivary glands.

### Topographic distribution of the broad histological types of head and neck cancer in the maxillofacial area

Carcinomas and sarcomas of the maxillofacial region were commonest in the maxilla, while the highest number of lymphomas occurred in the mandible (Table 6). Other common sites for carcinomas were the salivary glands and palate, while the only other common site for sarcomas was the mandible. Lymphomas also occurred in the maxilla and tonsil. The lip was the site least affected by carcinomas, sarcomas and lymphomas. No lesion was indicated as involving the gingiva alone. In addition, no mesenchymal or haematological malignancies were found in the floor of the mouth, as seen in Table 6 below.

**Table 6:** Anatomical distribution of broad histological types of head and neck cancer within the maxillofacial region

ANATOMICAL SITE	HISTOLOGICAL TYPES			Total
		
	Carcinomas	Lymphomas	Sarcomas	
**Palate**	53(17.1%)	2(2.9%)	0	55
**Cheek**	19(6.1%)	1(1.4%)	5(9.4%)	25
**Tongue**	24(7.7%)	0	1(1.9%)	25
**Tonsil**	12(3.9%)	13(18.6%)	1(1.9%)	26
**FOM**	11(3.5%)	0	0	11
**Lip**	8(2.6%)	1(1.4%)	0	9
**Mandible**	43(13.9%)	26(37.1%)	16(30.2%)	85
**Maxilla**	63(20.3%)	22(31.4%)	20(37.7%)	105
**Salivary glands**	57(18.4%)	2(2.9%)	1(1.9%)	60
**Face**	20(6.5%)	3(4.3%)	9(17.0%)	32
**TOTAL**	310(100.0%)	70(100.0%)	53(100.0%)	433

KEY: FOM- FLOOR OF MOUTH

## Discussion

In this 19-year study, the frequency of malignant head and neck neoplasms was 62 cases per year, which is in agreement with a 15 year study of Adeyemi et al [7] from the same centre. However our figure is higher than previous Nigerian hospital based studies of head and neck cancer, which was reported as 31 cases from Obafemi Awolowo University in Ile-Ife, Nigeria [14], 47 cases from Jos University Teaching Hospital [15] and 38 cases from Lagos University Teaching Hospital [16]. The higher number seen at the University College Hospital, Ibadan could be due to the availability of facilities for multimodality management of head and neck cancer patients in the hospital as compared to some of the other centres [7].

A study in North America showed a relatively steady rise in cases of head and neck cancer from 1985-1994 [17].Our study however showed no regular pattern of increase or decrease in cancer cases. This discrepancy may be due to the failure of patients to present at hospitals in developing countries such as Nigeria because of lack of awareness and or lack of financial resources to cater for conventional medical therapy. An additional factor is the preference of many patients for non-orthodox medical care, which contributes to late presentation or complete lack of presentation, thereby distorting the true epidemiological picture [18].

The male to female ratio of 1.9:1 in this study, is in agreement with 1:1 to 2.3:1 reported by Lilly-Tariah *et al *[19] in which a meta-analysis review of twenty-seven relevant published articles on head and neck cancers in Nigeria from 1968 to 2008 was undertaken. Further supporting findings from the present study, are male: female ratios of 1.7:1 in a six year review by Abuidris *et al *[20] in central Sudan and a 2.4:1 ratio in a 13 year Japanese study [21]. Furthermore, a 19 year Chinese study reported a male to female ratio of 2.4:1 [22]. These studies, which consider all head and neck malignancies together, support a male preponderance but separate consideration of each group of malignant lesions may give a clearer picture of gender distribution.

The overall age range of 1 year to 98 years in this study is in agreement with the meta-analysis of related Nigerian studies where a range of 9 months to over 80 years was reported [19]. Head and neck malignancies generally occurred least frequently within the first 14 years of life and displayed a gradual increase until it peaked in the 45-64 years range (36.4%). This peak is slightly higher than the 3^rd ^to 6^th ^decades (20-59 years) reported by Lilly-Tariah [19] who included in their study, thyroid and ocular malignancies, which could be relatively high in children. Overall mean age of 43.9 years (SD ± 19.3) for patients in this study is comparable to 48.8 years reported by Abuidris [20]. This may have been influenced by the fact that both studies had a large proportion of squamous cell carcinomas, which are known to peak in the 5^th ^decade (40-49 years) [23].

The general topography in this study indicated that the upper respiratory tract (43.2%) was the most common site affected by head and neck cancers. This finding is similar to a report on the overall pattern of head and neck cancers from different regions of Nigeria, in which nasopharynx, nose and larynx were the three most common sites (in descending order) [19]. In contrast Amusa *et al *[14] reported the oral cavity as the most common site in Ile-Ife, Nigeria (south-west) accounting for 36.8% of cases. In this present study however the oral cavity was the third most common site (12.5%) after the maxillofacial bones (20.5%). The reason for the discrepancy between the Ibadan and Ile-Ife study is not clear since both centres are in the South West of Nigeria and are exposed to similar diets and environmental factors. A study performed in central Sudan found the oral cavity to be the fourth most common site (10.5%) after the upper respiratory tract (72.7%) which was the commonest site [20].

In an analysis of over 19,400 patients with malignant head and neck tumours in Guangxi province of China, the most frequently involved sites were the nasopharynx followed by the mouth, maxillofacial regions and the neck [22]. Consumption of preserved food particularly salted fish has been implicated in nasopharyngeal cancer in China [24]. Findings in this study are in consonance with most other studies concerning the most commonly affected site in head and neck malignancies. Wood smoke in ill-ventilated houses in Africa, wood dust and Epstein Barr virus infection have been suggested as possible predisposing factors in Africans [25] and may account for the findings in this study.

Observation in the present study that most of the tumours seen in the 1-14 years age range were lymphomas (54.1%) are similar to the report by Bailey *et al *[26] which reported lymphomas as constituting 57% of head and neck malignancies in children. Further observation that lymphomas involved mainly the lymph nodes (39.7%), is consistent with the report of Hoffman *et al *[17] who also found that lymph nodes of the head and neck were the most common sites for lymphomas.

Sarcomas of the head and neck had an overall male: female ratio of 1.5:1. This finding is similar to the 1.3:1 male: female ratio reported by Adebayo *et al *[27] in Kaduna state, Nigeria. However the Memorial Sloan-Kettering Cancer Centre study [28] reported a male: female ratio of about 1:1.

The neuroendocrine malignancies seen in this study had a male: female ratio of 1:1, which agrees with the male: female of 1:1 reported in a study by Monroe *et al *[29] and is close to the 1.3:1 reported in a study in the USA [30]. However, it is at variance with the female to male ratio of 2.3:1 observed by Castelnuovo *et al *[31]. It is pertinent to mention that neuroendocrine carcinomas are rare.

Separate consideration of the oro-facial complex (maxillofacial bones and oral cavity) in this study found that the most common sites were the maxilla (24.3%), mandible (19.7%) and salivary glands (13.9%). Intraorally, the palate was the most common site (12.7%) followed by the tonsils and the cheek. This is in agreement with findings by Lawoyin *et al *[32], also from Ibadan, who reported that the palate was the most common intraoral site, but is at variance with a report by Odukoya *et al *[33] from Lagos in which the mandibular gingiva, maxillary gingiva and hard palate were the most common intraoral sites (in descending order). Other studies from Nigeria showed the tongue, palate and mandibular alveolus as the most commonly affected sites (in descending order) [34,35]. In South East Asia, the buccal mucosa and retromolar areas were the most prone areas [36]. Ugboko *et al *[37] from Ile-Ife, Nigeria reported the alveolus (29.6%) as the most common intraoral site. It is our thought that malignant lesions which may have originated in the gingiva and subsequently invaded the alveolus of patients in our study were diagnosed as maxillary or mandibular cancers, due to late presentation.

The broad histological types of malignancies in the present study were carcinomas (73.4%), lymphomas (17.5%), sarcomas (8.9%) and neuroendocrine tumours (0.2%). This contrasted favourably with findings by Adeyemi *et al *[7] that reported carcinomas (71.7%), lymphomas (20.4%) and sarcomas (7.9%) to be the major categories. Another study in Plateau state, Nigeria found that carcinomas predominated over sarcomas with lymphomas featuring in between [38]. In contrast Amusa *et al *[14] in a ten year review on the pattern of head and neck malignant tumours reported lymphomas (40.3%) as the predominant histological type followed by squamous cell carcinomas (25.3%), sarcomas (2.6%) and other minor variants (31.9%). The consideration of squamous cell carcinoma as the only epithelial malignancy in their study may have resulted in the perceptible dominance of lymphomas.

In the National Cancer Data Base, which is limited to 50 states and the District of Columbia, United States of America, Hoffman [17] reported 295,022 cases of head and neck cancers of which carcinomas accounted for 75.2% while lymphomas constituted 15.1%. This result is also similar to the present study except that the criteria for inclusion of cancers vary and lesions like sarcomas and neuroendocrine tumours were not clearly specified.

It was noted in this study that carcinomas increased in incidence while lymphomas reduced from 2002-2008. A report by the Surveillance Epidemiology and End Results [39] programme noted a slight gradual increase when all head and neck cancers are considered together from 1985-2005 [39]. The increase in carcinomas noted in the present study may be due to the increasing incidence of head and neck cancers in women consequent to an increasing exposure to risk factors [40,41], while the decrease in lymphomas may actually reflect the decreasing incidence of Burkitt's lymphoma in Nigeria which may be ascribed to improved living conditions and better management of malaria [42].

From the 1192 malignant cases of head and neck malignant tumours investigated in this study, 142 (11.9%) were poorly differentiated/undifferentiated while the remaining 1050 (88.1%) were differentiated, giving a ratio of undifferentiated to differentiated tumours in this study as 1:7.4, which is higher than 1:9 computed from the study by Vege *et al *[43]. Relatively higher prevalence of poorly differentiated cancers in Africans has been reported [44].

## Conclusion

The clinico-pathological summary of head and neck malignancies in western Nigeria is not different from profiles in other parts of the world. Malignancies of epithelial lineage are more common than other lineages in the head and neck whereas neuroendocrine tumours of the head and neck are rare. It is expedient to conduct this sort of study periodically to monitor the changing trends of head and neck cancer so that apt attention can be accorded the predominant type and changes can be investigated to know if a new carcinogen has been introduced to the environment.

## Competing interests

'The author(s) declare that they have no competing interests'

## Authors' contributions

AO, AO and JO were involved in the conception and design of the study. AO acquired the data. AO and BF participated in the analysis and interpretation of the data. BF, EEU and B were involved in drafting the manuscript. AO and EEU revised the manuscript. All authors read and approved the final manuscript.
